# The R263K mutation in HIV integrase that is selected by dolutegravir may actually prevent clinically relevant resistance to this compound

**DOI:** 10.7448/IAS.17.4.19518

**Published:** 2014-11-02

**Authors:** Mark Wainberg, Kaitlin Anstett, Thibault Mesplede, Peter Quashie, Yingshan Han, Maureen Oliveira

**Affiliations:** McGill University AIDS Centre, Jewish General Hospital, Montreal, Canada

## Abstract

**Introduction:**

Drug resistance against dolutegravir (DTG) or the nucleosides with which it has been co-administered has never been observed in patients receiving this drug in first-line therapy. In contrast, a R263K mutation that confers low-level resistance (3–4 fold) to DTG has been selected by DTG in culture. Our group has ascribed the absence of resistance to DTG to the high fitness cost exacted by the R263K mutation and an inability of HIV to generate compensatory mutations.

**Materials and Methods:**

We generated recombinant integrase enzymes and viruses containing various combinations of mutations and studied these enzymatically and in culture. We also selected for resistance against raltegravir (RAL) using viruses containing the R263K mutation.

**Results:**

The R263K mutation alone conferred an approximate 3-fold level of resistance to DTG and a 40% loss in viral replicative capacity and recombinant integrase activity. Secondary mutations selected at positions H51Y or E138K did not individually affect either enzyme activity or DTG resistance, but the combination of R263K together with H51Y or E138K increased DTG resistance to about 7-fold accompanied by a ≈75% loss in each of viral replication capacity, and both *in vitro* and *in vivo* integrase activity. Conversely, combinations of R263K together with multiple resistance mutations for RAL and/or EVG at positions 92,143, 148 and 155 resulted in even further diminished enzymatic activity that may be incompatible with viral survival. Modelling of the 3-dimensional structure of integrase suggests that R263K is located in a region that may not permit further mutagenesis if secondary mutations at H51Y or E138K are also present. Moreover, integrase that contains R263K together with substitutions at positions 92, 143, 148 and 155 may be enzymatically inactive. The use of the R263K-containing virus to select for resistance to RAL led to the appearance of RAL-containing mutations but the loss of R263K.

**Conclusions:**

Secondary mutations to R263K following selection with DTG have all led to diminished viral and enzymatic fitness, helping to explain why resistance to DTG in previously drug-naïve subjects has never been observed. The use of DTG in first-line therapy may prevent the facile development of drug resistance and help to forestall ongoing HIV transmission.

